# Weakly supervised learning analysis of Aβ plaque distribution in the whole rat brain

**DOI:** 10.3389/fnins.2022.1097019

**Published:** 2023-01-19

**Authors:** Zhiyi Chen, Weijie Zheng, Keliang Pang, Debin Xia, Lingxiao Guo, Xuejin Chen, Feng Wu, Hao Wang

**Affiliations:** ^1^National Engineering Laboratory for Brain-Inspired Intelligence Technology and Application, School of Information Science and Technology, University of Science and Technology of China, Hefei, China; ^2^Institute of Artificial Intelligence, Hefei Comprehensive National Science Center, Hefei, China; ^3^AHU-IAI AI Joint Laboratory, Anhui University, Hefei, China; ^4^School of Pharmaceutical Sciences, IDG/McGovern Institute for Brain Research, Tsinghua University-Peking University Joint Center for Life Sciences, Tsinghua University, Beijing, China

**Keywords:** Aβ plaque, rat brain, deep learning, light sheet microscopy, weakly supervised learning segmentation, quantitative analysis

## Abstract

Alzheimer’s disease (AD) is a great challenge for the world and hardly to be cured, partly because of the lack of animal models that fully mimic pathological progress. Recently, a rat model exhibiting the most pathological symptoms of AD has been reported. However, high-resolution imaging and accurate quantification of beta-amyloid (Aβ) plaques in the whole rat brain have not been fulfilled due to substantial technical challenges. In this paper, a high-efficiency data analysis pipeline is proposed to quantify Aβ plaques in whole rat brain through several terabytes of image data acquired by a high-speed volumetric imaging approach we have developed previously. A novel segmentation framework applying a high-performance weakly supervised learning method which can dramatically reduce the human labeling consumption is described in this study. The effectiveness of our segmentation framework is validated with different metrics. The segmented Aβ plaques were mapped to a standard rat brain atlas for quantitative analysis of the Aβ distribution in each brain area. This pipeline may also be applied to the segmentation and accurate quantification of other non-specific morphology objects.

## 1. Introduction

Alzheimer’s disease (AD) is a progressive degenerative disease of the central nervous system that causes cognitive decline and extensive neuronal death (Barage and Sonawane, 2015). It is one of the most common dementias among elderly individuals. Modern society has to tackle the heavy burden of aging as a result of AD. Extensive explorations in developing drugs and therapy for AD have failed. One of the major reasons is the lack of animal models that could fully mimic the pathological features of AD (Scearce-Levie et al., 2020). The most prevalently used animal models in AD studies are transgenic mice overexpressing amyloid precursor protein (*APP*), including PDAPP and Tg2576 (Games et al., 1995; Hsiao et al., 1996). However, the non-physiological and ectopic expression of *APP* in transgenic mice has never been demonstrated in AD patients. Other transgenic mice with both *APP* and *PSEN1* mutations, including *APPswe/PS1M46L*, *APPswe/PSEN1dE9*, and 5× *FAD* mice, have been widely used in AD studies. However, these animal models showed little neuronal loss, no tau pathology, or even provoked beta-amyloid (Aβ) pathology in ectopic brain areas that were not present in human AD patients. Compared to mice, the physiology and behavior of rats are more similar to those of human beings. Recently, a new AD rat model was developed by knock-in of App with CRISPR/Cas9, exhibiting most of the pathological features of AD (Pang et al., 2022). This model rat shows both Aβ plaques and tau pathology in the brain, which is the first-ever rodent model demonstrating these two main deficits.

Among many pathological features of AD, the deposition of Aβ is one of the main phenotypes. The density and distribution of Aβ plaques are crucial indicators of disease development. Quantitative analysis of Aβ plaques is critical for studying the spatial-temporal origin and evolution of the disease (Long et al., 2019). Modern neuroimaging techniques such as computed tomography (CT), positron emission tomography (PET) imaging (Koychev et al., 2020), or magnetic resonance imaging (MRI) (Sheikh-Bahaei et al., 2017), are widely used in quantifying Aβ accumulation in the brain. These non-invasive imaging methods have been used for clinical diagnosis. However, due to the low resolution and specificity, the diagnostic results are not accurate. *Ex vivo* studies with model animals could use immunostaining and microscopic imaging of continuous brain slices. However, the quantitative analysis of Aβ plaques on whole-brain image datasets remains a great challenge, owing to the high consumption of money and time during the acquisition and analysis of the whole-brain Aβ imaging dataset. With the development of high-speed volumetric microscopic imaging techniques (Gong et al., 2016; Wang et al., 2019), rapid microscopic 3D imaging of the whole mouse brain can be achieved in days or hours. A previous study proposed a framework for Aβ staining, imaging, and quantification of the whole mouse brain (Long et al., 2019), but the morphological characterization of Aβ in the whole rat brain has not yet been investigated. Meanwhile, the imaging speed was relatively slow, which makes it quite time-consuming for whole rat brain imaging.

Using the high-speed volumetric imaging method we have developed (Wang et al., 2019), imaging of the whole rat brain at micrometer resolution can be completed within 4 h. This approach will generate approximately 8 TB raw data at a 1 μm × 1 μm × 3.5 μm voxel size with an intact adult rat brain. A highly efficient pipeline for automatic segmentation and quantitative analysis of whole-brain Aβ plaques is needed.

Segmentation is a crucial step in assessing the accurate brain-wide distribution of Aβ plaques. In traditional segmentation methods, features and parameters need to be set manually, which is not suitable for automatic and accurate segmentation of morphologically diverse Aβ plaques in whole-brain 3D microscopic images (Berg et al., 2019; Chen et al., 2020). With the rapid development of deep learning in recent years, segmentation methods based on fully supervised learning have been improved significantly, which requires large human labeling costs (Ronneberger et al., 2015; Chen et al., 2018). To reduce the cost of manual annotation, weakly supervised learning with weak annotations is more appropriate for biological microscopic images (Jia et al., 2017; Zhao et al., 2018). Here, we propose a segmentation method based on weakly supervised learning, only requiring object-level annotations, in which the cost of labeling is 1/15 of the pixel-level annotations. We adopt the high-resolution network (HRNet) as the feature extractor (Wang et al., 2020) and deploy it into a multi-stage object detection framework Faster-RCNN (Ren et al., 2015). Subsequently, extra visual cues using peak response mapping (Zhou et al., 2018) are provided for segmentation with the 2D-OTSU algorithm (Zhang and Hu, 2008) in post-processing. In addition, a pre-processing method is provided according to the characteristics of the image dataset, which can reduce the attenuation of the signal intensity caused by the thickness of tissue slices and improve the signal-to-noise ratio (SNR).

To quantitatively analyze the distribution of plaques in different brain areas, we registered the 3D whole-brain dataset to the Waxholm Space Sprague Dawley (WHS-SD) rat brain atlas (Papp et al., 2014). Utilizing the deformation field produced during registration to the Aβ binary mask of the whole brain, all brain areas of the imaged dataset were aligned to the standard brain map for subsequent quantification of Aβ plaques.

## 2. Materials and methods

### 2.1. Sample preparation

**FIGURE 1:**
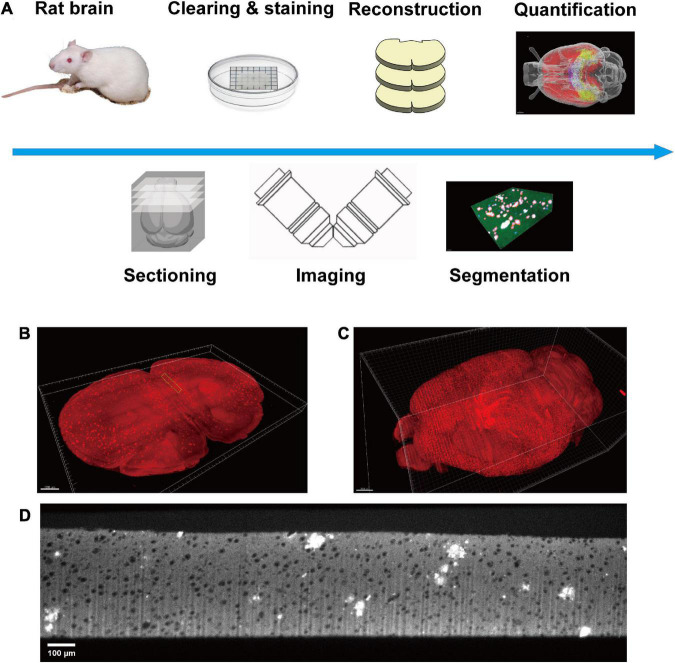
Diagram of this study. **(A)** The pipeline of the whole process, including sample preparation, data acquisition, plaque segmentation, and quantification analysis. **(B)** Single slice imaged with micron resolution. **(C)** The 3D-reconstructed whole rat brain. **(D)** The raw image of a section in panel **(B)**, green boxed.

The rat brain was prepared as reported previously (Pang et al., 2022). Briefly, adult animals were sacrificed by transcardial perfusion of 40 mL 1× phosphate buffered saline (PBS) and 40 mL 4% paraformaldehyde (PFA) successively. Subsequently, the sample preparation is followed by the Volumetric Imaging with Synchronized on-the-fly-scan and Readout (VISoR) imaging procedure (Wang et al., 2019). The sample was transferred into ice-cold 4% hydrogel monomer solution (HMS) for post-fixation at 4 degrees for 48 h. After post-fixation, the 4% HMS was replaced with a mixture of 15 mL 4% HMS and 15 mL 20% bovine serum albumin (BSA). The solution was degassed in a vacuum pump for 20 min with ice surrounding the centrifuge tubes. The sample was polymerized at 37°C for 4 h and rinsed with PBS three times to remove residual reagents. Next, the sample was sectioned into 300 μm thick slices. All slices were cleared with 4% sodium dodecyl sulfate (SDS) solution at 37°C for 24 h with gentle shaking. The slices were washed with PBS three times, 1 h for each round. The slices were immune-stained with anti-Aβ primary antibody (Biolegend No. 803002, 1:500 dilution with PBS) for 24 h at room temperature and washed with PBS for three times, 1 h for each round. The secondary antibody (JacksonImmuno Research No. 715-545-150, 1:200 dilution with PBS) was applied for 6 h at room temperature with gentle shaking and washed out with PBS for three times, 1 h per round. Finally, all slices were mounted on a customized slide (100 mm × 100 mm) for imaging (Figure 1A).

### 2.2. Data acquisition

A modified VISoR microscope described in a previous study was used for all data collection (Xu et al., 2021). Two-channel imaging was carried out successively with 488 and 552 nm excitation. The emission light was collected through an Olympus 10 × 0.3 NA water immersion objective and filtered with bandpass filters (520/40, 600/50, from Semrock). Images were collected with a sCMOS camera (Flash 4.0 v3, Hamamatsu) (Figure 1A). All data were collected with a pixel resolution of 1 μm × 1 μm × 3.5 μm. The collected images were reconstructed into a dual channel whole rat brain for further segmentation and quantification (Figures 1B–D). The 3D reconstruction of the whole brain imaging dataset was the same as our previous study (Wang et al., 2019). Briefly, it mainly contains four steps. (i) Flattening the upper and lower surfaces of each brain section. (ii) Detecting the edges of adjacent sections and extracting the correspondences between two opposing surfaces. (iii) Morphing the correspondences of each section for limiting the morphological errors. (iv) Warping each section with the extracted and morphed correspondences.

### 2.3. Data preprocessing

A preprocessing pipeline of the dataset is presented to calibrate the brightness of serial brain sections as well as to enhance the signal-to-noise ratio. This step is crucial for the overall success of network training and testing. Two preprocessing strategies were applied successively:

**Brightness calibration of the brain slices.** The brightness of the imaging channel varies at different depths of the slices. Two reasons contribute most: (a) the difference in antibody concentration at different depths and (b) excitation light absorption by tissue through the optical propagation path. The brightness over *z*-axis was measured and automatically calibrated (Supplementary Figures 1A, B). Specifically, the corrected brightness of image stacks can be formulated by:


(1) $$I\,{\left(z\right)}^{{}^{\prime }}=\frac{I\,\left(z\right)}{I_{m\,e\,a\,n}\,\left(z\right)/I_{m\,e\,a\,n}\,\left(z_{m\,i\,d}\right)}$$

*I*(⋅) represent the intensity of each pixel. *I*_*mean*_(⋅) indicates the mean intensity of each image slice. *z* means the slice index of the image stacks. The non-brain-slice pixels are excluded while calculating the mean intensity.

**Signal-to-noise ratio (SNR) enhancement.** As the Aβ plaques were labeled by immunofluorescent staining, the intensity of the fluorescence signal was relatively weak for image segmentation. The low SNR images will lead to poor performance in network training for image segmentation. To further enhance the segmentation performance, 3D Gaussian blur (σ_*x*,*y*,*z*_ = 2 for saving more details) was used to improve the SNR of all brain slices.

Due to the large size of 3D image stacks and the limitation of computer memory, we partitioned the image stacks into image blocks with size of 256 × 256 × 75. Image blocks having less than 1% brain tissue pixels were excluded for network training and testing.

### 2.4. HRNet structure

Segmentation is a position-sensitive computer vision task. Due to the high density and small size of Aβ plaques in the whole rat brain, over-down-sampling of training data in the neural network will lead to the missing and position offset of small objects. The parallel structure of HRNet can combine high-resolution features with high-level semantic information. The high-resolution representations learned from the HRNet are not only spatially precise but also semantically strong.

**FIGURE 2:**
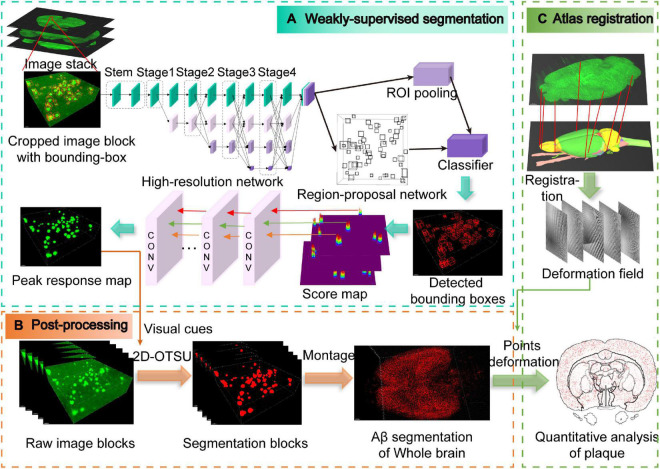
Schematic illustration of the entire analysis pipeline. **(A)** Weakly supervised segmentation with the high-resolution network. The pipeline is modified from the Faster-RCNN framework, and the peak response mapping method is embedded in our framework for extracting additional visual cues for subsequent segmentation. Note that only weak labels (bounding boxes) are required in our pipeline, which are shown as red boxes in the cropped image block. **(B)** Post-processing for obtaining whole-brain Aβ segmentation masks. **(C)** Obtaining the deformation field from the atlas registration process and then utilizing it to the segmentation masks from panel **(B)** for quantitative analysis of whole-brain plaques.

The HRNet is connected in parallel (Figure 2A), which consists of parallel branches with high-to-low resolution. The resolution of the rth branch is 1/(2*^r–1^*) of the resolution of the first stream, while the channel number is 2*^r–1^* of the first stream.

The basic module used in each subnetwork contains two 3 × 3 convolutional kernels where each kernel is followed by batch normalization (BN) and rectified linear unit (ReLU). Skip connection is used to connect the input and output of the module (Supplementary Figure 2A). The connection between different stages consists of transition modules and fusion modules (Supplementary Figure 2C), which are applied to exchange information between multi-resolution layers. Two output modes are provided (Supplementary Figure 2B): (i) HR-keep mode only outputs the high-resolution representation computed from the high-resolution convolution stream, (ii) HR-fuse mode combines the representations from all the high-to-low resolution parallel streams.

### 2.5. Weakly supervised learning with object-level labels

The training of neural networks is usually fully supervised and requires a large number of manual annotations, especially for image segmentation which requires pixel-level annotation. For 3D microscopic biomedical images, annotation requires extensive professional knowledge guidance, which undoubtedly leads to high expenses of money and time. In this paper, object-level weak annotations are used for pixel-level image segmentation tasks (Figure 2A). Specifically, we train the object detection framework with bounding box annotations and then segmentation is carried out by post-processing methods (Figure 2B and Supplementary Video 1).

Faster-RCNN (Ren et al., 2015) is a widely used object detection framework that usually consists of conv-body, region-proposal network (RPN), RoI-pooling, and classifier. In this work, a modified 3D form Faster-RCNN is used for detecting Aβ plaques in the volumetric image dataset. High-to-low resolution features of the 3D images are extracted by HRNet. After that, the features are input into the RPN and classifier to obtain the predicted bounding-box map. In Faster-RCNN, RPN depends on the default anchor settings, and we set the anchor size to fit the size range of Aβ plaques.

Subsequently, peak response mapping (PRM) (Zhou et al., 2018) is used to obtain additional visual cues to improve the segmentation performance (Figure 2A). The main idea of PRM is to generate a peak-response map by stimulating peaks in the class-aware map. Then, the most informative regions of each plaque are identified and mapped by the back-propagated peaks. Score maps generated from RPN in the Faster-RCNN framework can be regarded as a class of peak correspondence maps related to location (Dong et al., 2019). Therefore, we assume that peaks in the score map represent strong visual cues for the objects. The peak is back-propagated while the score map is sent to the classifier for further classification. The peak back-propagation can be interpreted as a random walk process. Each location in the bottom layer’s top-down relevance is formulated as its probability of being visited by the walker.

Consider a convolution layer with a filter size *s*×*h*×*w*. *I*_*ijk*_ and *O*_*pqt*_ are the spatial locations of the input and output feature maps, respectively. The visiting probability *P*(*I*_*ijk*_) can be formulated by:


(2) $$P\left(I_{i\,j\,k}\right)=\sum_{p=i-\frac{s}{2}}^{i+\frac{s}{2}}\sum_{q=j-\frac{h}{2}}^{j+\frac{h}{2}}\sum_{t=k-\frac{w}{2}}^{k+\frac{w}{2}}P\left(I_{i\,j\,k}|O_{p\,q\,t}\right)\times P\left(O_{p\,q\,t}\right)$$

where the transition probability is formulated as:


(3) $$P\left(I_{i\,j\,k}|O_{p\,q\,t}\right)=Z_{p\,q\,t}\times {\hat{I}}_{i\,j\,k}ReLU\left(W_{\left(i-p\right)\,\left(j-q\right)\,\left(k-t\right)}\right)$$

${\hat{I}}_{i\,j\,k}$ is the bottom-up activation value during the forward process. *ReLU*(*W*_(*i*−*p*)(*j*−*q*)(*k*−*t*)_) means excluding negative weights as they are not helpful in improving the output response. *Z*_*pqt*_ is a factor to ensure ∑_*p*,*q*,*t*_ *I*_*ijk*_|*O*_*pqt*_ = 1. Note that PRM only needs to be activated during testing rather than training.

Finally, we adopt an advanced 2D-OTSU (Zhang and Hu, 2008) algorithm for segmentation (Figure 2B). Unlike the original algorithm that uses handcrafted features as the second-dimension input, alternatively, we use the peak response map extracted from the neural network, which provides additional information in addition to grayscale intensity.

### 2.6. Rat brain atlas registration and quantitative analysis

To quantitatively analyze the distribution of plaques in different brain regions, the 3D reconstructed rat brain needs to be registered to a rat brain atlas reference (Figure 2C). The WHS-SD rat brain atlas (Papp et al., 2014) is one of the most commonly used digital rat brain atlases. In this paper, we utilized atlas version 2 from public resources^1^, which has 80 brain areas. We modified the images to remove the skull structures in the T2* template images, leaving only the brain structures.

The reconstructed 3D rat brain image dataset was registered to the WHS-SD brain atlas. Specifically, we used the WHS-SD rat brain template, annotation file, and atlas file provided by the public resources (see text footnote 1). The images were then converted to SimpleITK (Lowekamp et al., 2013) format to achieve fast alignment with the Elastix (Klein et al., 2009) toolbox. We adopted the non-rigid B-spline as the transform model. Mutual information (MI) and rigidity penalty was used as metrics for the similarity measure. The deformation field was optimized globally by using the stochastic gradient descent (SGD) algorithm. Order of B-spline interpolation was set to 3. Subsequently, a neuroanatomy expert was engaged to fine-tune the borderlines of all brain areas.

### 2.7. Evaluation metrics

To comprehensively evaluate the accuracy of our weakly supervised segmentation method, we use three metrics for quantitative analysis, including the Dice score (DSC), Sensitivity (SST), and Hausdorff distance (HD). These metrics are defined as follows:


(4) $$D\,S\,C=\frac{2\,T\,P}{2\,T\,P+F\,P+F\,N}$$

(5) $$S\,S\,T=\frac{T\,P}{T\,P+F\,N}$$

where TP denotes the true positive of the predicted pixels. FP denotes the false positive. FN denotes the false negative.


(6) $$d_{H}\,\left(X,Y\right)=m\,a\,x\,\left\{s\,u\,p_{x\in X}\,i\,n\,f_{y\in Y}\,d\,\left(x,y\right),s\,u\,p_{y\in Y}\,i\,n\,f_{x\in X}\,d\,\left(x,y\right)\right\}$$

where *X*,*Y* denote the ground-truth pixel set and segmentation pixel set in the image segmentation task. *sup* represents the supremum. *inf* represents the infimum. *d*(⋅) is the distance metric.

Dice score is sensitive to interior pixels. SST represents the omission rate. HD is sensitive to boundary pixels. Note that 95% Hausdorff distance (HD95) is used here to remove the effect of minimal outliers.

## 3. Result

### 3.1. Weakly supervised segmentation results

We randomly selected 30 cropped blocks from the cerebral cortex as training data, and each block size was 256 × 256 × 75. Two experts were engaged to annotate images with bounding boxes. In addition, 28 blocks were randomly selected from different brain areas in three categories: cerebral cortex (Cortex), hippocampus (Hippo), and other brain areas (Other) as test data. All test image blocks were labeled with pixel-level annotations to evaluate the performance of our weakly supervised segmentation method. The preprocessing method was applied to each image block. All experiments were trained and tested with the PyTorch framework on a workstation with one NVIDIA Tesla V100S and 768 GB RAM. We carefully set the anchor sizes in range of [6,28] and the stride of RPN as 4. We used the SGD optimizer with the learning rate 0.01, the weight decay of 0.0001. The training process was terminated within 3,000 iterations.

**FIGURE 3:**
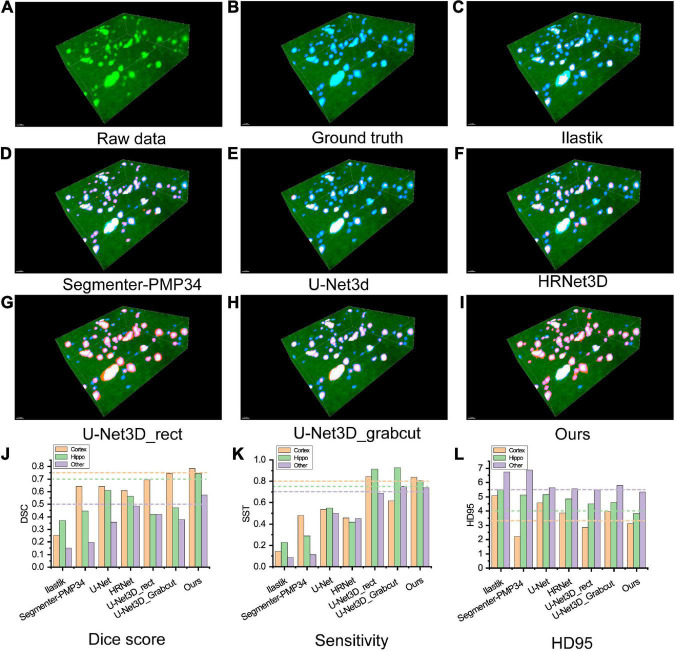
**(A–I)** Visualization of comparing the results of different segmentation methods on an image block of test set. Green: raw image, blue: ground truth, red: segmentation result. **(A)** Raw image. **(B)** Ground truth. **(C,D)** Classical segmentation methods with the handcrafted label, **(C)** ilastik, **(D)** segmenter-PMP34. **(E,F)** Fully supervised methods, **(E)** U-Net3d, **(F)** HRNet3D. **(G,H)** Weakly supervised methods labeled with rectangles and Grabcuts, respectively. **(I)** Our method. **(J–L)** Metrics of segmentation performance. **(J)** Dice score, **(K)** Sensitivity, **(L)** HD95. Note that HD95 indicates the distance between the segmentation mask and the ground truth, lower score indicates better performance.

We compared the proposed framework with widely used segmentation methods, including (a) traditional methods based on artificial features, such as Ilastik (Berg et al., 2019) and Segmenter-PMP34 (Chen et al., 2020); (b) fully supervised learning methods, such as U-Net (Ronneberger et al., 2015) and HRNet; and (c) weakly supervised learning methods with different weak labels, such as U-Net3D_rect and U-Net3D_grabcut (Khoreva et al., 2017). Different segmentation methods showed discernible results (Figures 3A–I). The evaluation metrics of different methods were calculated in distinct brain areas (Figures 3J–L and Supplementary Table 1).

Ilastik is a machine learning method based on the random forest algorithm. The user explicitly marks the features manually and applies batch processing to segment other images. However, this grayscale feature-based method showed the worst robustness through the whole brain, resulting in the poorest performance in the experiments. In addition, the computational cost of Ilastik is the highest due to CPU-based implementation. Segmenter_PMP34 is a conventional segmentation pipeline consists of Min-max intensity normalization, 2D Gaussian smoothing, 2D spot filter, watershed algorithm and size filter, which requires manual parameter adjustment while all methods in this experiment were fully automatic. For fairness, the parameters were adjusted in a cerebral cortex slice to reach the best performance and then applied to other brain slices. The metrics of the Hippo and other regions were significantly lower than our method. Interestingly, in the cerebral cortex region, the HD95 of Segmenter_PMP34 was better than our method but severely sacrificed SST indicating that many object pixels had not been detected.

To train the fully supervised network, two experts relabeled the training dataset with pixel-level labels. The U-Net architecture consists of an encoding and a decoding part. Encoding part repeatedly applies two 3 ×3 convolutional layers, each followed by a batch normalization layer and a ReLU. At each down-sampling step, the number of features is doubled. Decoding part recovers the original size by up-sampling the feature map. Every step of up-sampling consists of an up-sample layer that halves the number of features, and two 3 ×3 convolutional layers, each followed by a batch normalization layer and a ReLU. The final layer is a softmax layer. The HRNet architecture is the same as which used in our methods and HR-fuse mode is utilized for the best performance. We used the SGD optimizer with the learning rate 0.01, the weight decay of 0.0001 and momentum of 0.9. However, fully supervised segmentation requires a large number of pixel-level labeled image datasets. U-Net and HRNet produced poor segmentation results, partly because the training set was relatively small. All metrics were lower than our method, especially in SST.

We generated pixel-level labels through weakly labeled bounding boxes and then trained the semantic segmentation network iteratively (Khoreva et al., 2017). U-Net_3D_rect treats the pixels inside the 3D bounding box as pseudo labels. U-Net_3D_grabcut utilizes the GrabCut (Rother et al., 2004) algorithm inside the 3D bounding box to obtain the initial pseudo labels. We used these two pseudo labels to train the U-Net, respectively. The network architecture and parameter settings were the same as fully supervised network. The SST of these two methods was slightly better than our method, but Dice and HD95 were worse than ours as these methods treated too many background pixels as object pixels.

Furthermore, we clustered the plaques into three different categories according to their volumes, (a) small (<300 voxels), (b) medium (300–1,500 voxels), and (c) big (>1,500 voxels). The voxel size used here is 4 μm × 4 μm × 4 μm. We evaluated the performance of our method to analyze plaques of different sizes (Supplementary Figure 3 and Supplementary Tables 3, 4).

Our method only needed the bounding boxes as the weak labels. Almost all metrics achieved state-of-the-art performance. In addition, the ablation study was completed to verify the effectiveness of each module in our framework (Supplementary Table 2), which proved that HRNet (including fuse output mode), PRM, and 2D-OTSU all contributed to improving the segmentation performance.

### 3.2. Quantification of Aβ plaques in the whole rat brain

**FIGURE 4:**
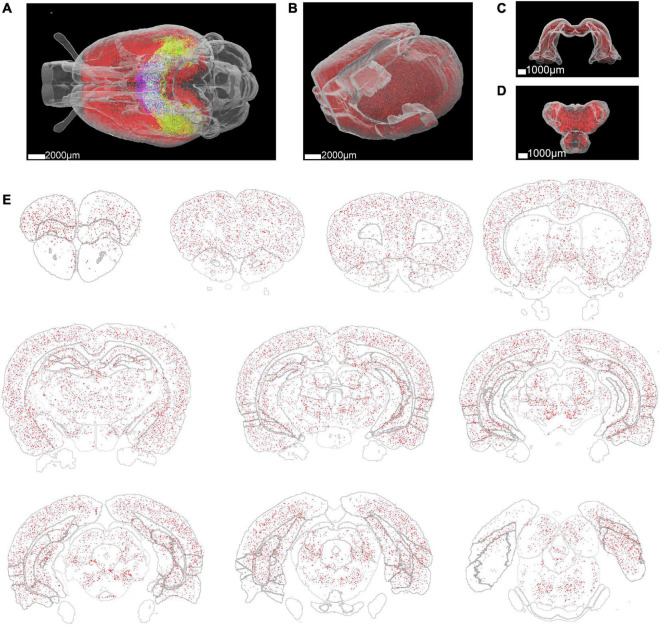
Whole rat brain rendering of segmented Aβ plaques. **(A)** The Aβ plaque distribution in the whole brain of a 3-month-old rat. Different colors represent the Aβ plaque distribution in different brain regions. Red: cortex, green: hippocampus, blue: thalamus. **(B–D)** Enlarged view of the Aβ plaque distribution in the cortex, hippocampus, and thalamus. **(E)** Aβ plaque distribution in 300 μm thick brain slices at different coronal planes.

**FIGURE 5:**
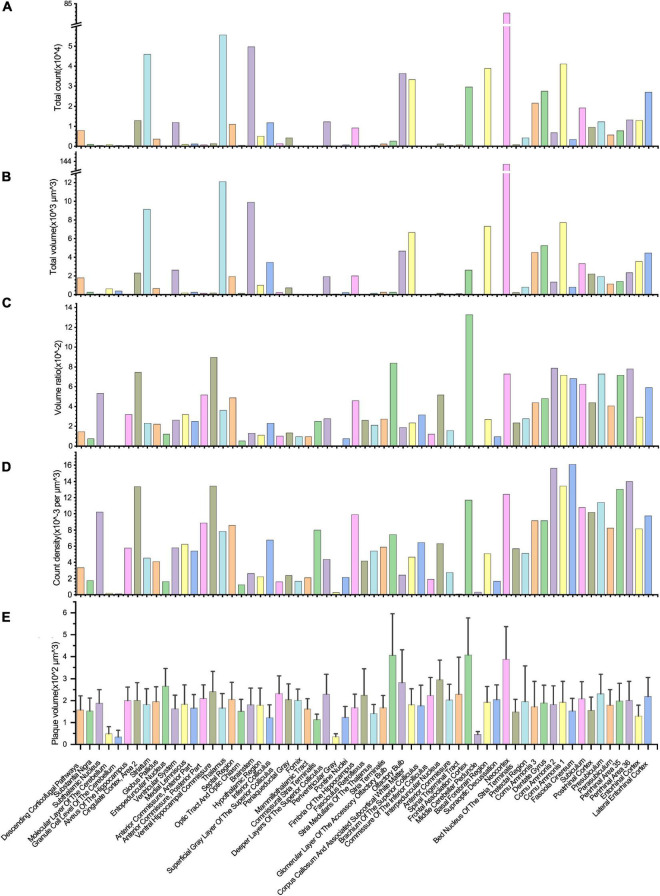
Quantitative analysis of the Aβ plaque distribution in different brain areas. **(A–D)** The total volume, total counts, volume ratio, and the count density of Aβ plaques in different brain areas. **(E)** The volume variation of Aβ plaques in distinct brain areas.

To quantitatively analyze the distribution of Aβ plaques in the whole rat brain, segmented image blocks were montaged into brain slices and then reconstructed into the whole brain (Supplementary Videos 2, 3). Then we utilized a registration process to align the whole brain to the WHS-SD rat brain atlas (Figure 2C). The implementation of the registration method was based on SimpleITK (Lowekamp et al., 2013) and Elastix toolbox (Klein et al., 2009), performed on a workstation with 384 GB of RAM. The initial deformation field was subsequently obtained and fine-tuned by an expert. After registration, we applied the deformation field to the Aβ whole-brain segmentation binary masks. 3D rendering was performed for visualization of Aβ plaque distribution in the whole rat brain of several brain areas, including the cortex, hippocampus, and thalamus (Figures 4A–D). Aβ plaque maximum intensity projection in the coronal plane from the olfactory bulb to the caudal end revealed plaque density diversity in different brain areas or nuclei (Figure 4E). Finally, the Aβ plaque distribution was accurately calculated based on segmentation and registration by 61 brain regions that excluded several brain areas from the original atlas which did not contribute to our quantitative analysis, such as nerves, decussations, and commissures (Figure 5).

Most plaques were found in the neocortex, followed by the thalamus, brainstem, striatum, and other brain regions (Figure 5A). Due to the lack of a high-resolution rat brain atlas, we could not analyze our segmentation results by more fine-grained hierarchical brain areas or subregions. The total volume of segmented plaques in each region was similar to the tendency of total plaque counts (Figure 5B). We also measured the volume ratio of total plaque volume to the brain region volume by each brain region, and found that the volume ratio is higher in the frontal association cortex than in others (Figure 5C). We further calculated the plaque count density and the average plaque volume in each brain region (Figures 5D, E). Very few vascular-associated amyloid depositions were found in the early stage AD rat brain imaging data. Since we do not intend to distinguish these vascular-associated amyloid depositions from non-vascular amyloid depositions by our deep learning-based analysis pipeline, the segmentation result involves all kinds of amyloids.

## 4. Discussion

In this study, we presented a pipeline for systematic quantitative analysis of whole rat brain Aβ plaque distribution. First, we applied the high-throughput volumetric imaging method VISoR to the newly developed AD rat model brain and acquired micron-resolution 3D images of Aβ plaques of the whole brain. Then, we developed a weakly supervised framework for the segmentation of Aβ plaques. Finally, we registered the segmentation results to the WHS-SD rat brain atlas for quantitative analysis of Aβ plaques in each brain area.

The high-throughput volumetric imaging method VISoR used in this study demonstrated fast tissue immunostaining and high-speed microscopic imaging of rat brains. Traditionally, whole rat brain imaging with the confocal microscope of serial cryo-sections may take months, not even to mention the high risk of losing 10 μm thick sections during experiments or reconstructing thousands of serial images into a complete brain. The VISoR method can image a 300 μm thick section by a single scan, which dramatically increases the thickness of the section from dozens of micrometers to hundreds of micrometers. Therefore, the section number of each brain required is significantly decreased, e.g., 85 slices for the brain of a 3-month-old rat. Since the thickness of each brain slice is 300 μm, it takes only 24 h for tissue clearing and following 39 h for immunostaining. This is the most efficient whole rat brain tissue clearing and immunostaining approach to our best knowledge. Tissue clearing of thick sections is the key for fast immunostaining and high-throughput whole brain imaging. Immunostaining is capable of labeling the diffuse plaques, while the direct staining with methoxy-X04 dye, the most commonly used staining method for Aβ plaques in previous studies, could only label the dense-core plaques (Whitesell et al., 2019). In addition, the thick slice provides higher stiffness and mechanical integrity, which makes the 3D dataset of serial slices much easier to be reconstructed into an intact brain. One major limitation of this method is that 300 μm thickness increased the difficulty of antibodies to penetrate uniformly through the whole slice in comparison to immunostaining on thin sections, e.g., most commonly used 10 μm cryo-sectioned slices. This could be further improved with more experiments on optimizing the conditions of tissue clearing and immunostaining. Another limitation is that mechanical sectioning method inevitably results in signal loss, we verified this by calculating the percentage of small plaques over total plaques between adjacent sections. The small plaques percentage in inter-sections is lower than inner-sections that means the missed detection of small plaques (Supplementary Figure 4). Our thick section method could reduce less miss-detection of small plaques than thin section. Since all slices were immune-stained under the same conditions, we observed a consistent signal intensity distribution through depth on all slices, which means we could calibrate the signal before any quantitative analysis. In addition, this high-throughput imaging method has already been successfully used in whole mouse brain and whole monkey brain imaging (Wang et al., 2019; Xu et al., 2021).

The high-dynamic-range volumetric microscopic fluorescence image is difficult to be segmented by traditional intensity-based algorithms. Deep neural networks could achieve better segmentation performance by learning texture features and semantic information of images. Meanwhile, the computation efficiency of deep learning-based methods implemented on the GPUs prevailed the most traditional methods implemented on CPUs. To achieve high-precision segmentation of Aβ plaques for quantitative analysis in the whole rat brain, this study proposes a weakly supervised segmentation framework based on HRNet and Faster-RCNN. To our knowledge, although HRNet and Faster-RCNN are widely used in diverse applications of artificial intelligence, there are few researchers using them in weakly labeled biomedical image segmentation tasks, especially in whole-brain 3D microscopic images of different biomarkers.

High-resolution network is the backbone for feature extraction in this framework, which connects the high-to-low resolution branches in parallel rather than in series, allowing high-resolution features to be maintained. This parallel structure can avoid the distortion of features during the up-sampling process in a serial structure such as U-Net. Therefore, the features are spatially precise. In addition, the repeated multi-resolution fusion modules make the high-to-low resolution features semantically strong. Furthermore, due to the high requirement of annotated image dataset of fully supervised learning method, we alternatively adopt the weakly supervised learning method, which only requires little human-labeling cost for network training and whole-brain Aβ plaque segmentation. Since Faster-RCNN is a multi-stage object detection network, our weakly supervised segmentation network would increase the computational complexity in comparison with other semantic segmentation networks which had been trained for classifying each pixel of the image. Peak response mapping introduces additional information into the network, with each peak representing a strong visual cue of the object. The peak response maps can also be used as the second-dimension input of the 2D-OTSU algorithm to improve the segmentation accuracy. However, the segmentation accuracy on small plaques (smaller than 6 pixels in diameter) is lower than medium and big plaques, as small object detection is a longstanding challenge in deep learning field (Supplementary Figure 3 and Supplementary Tables 3, 4).

Previous studies have reported several Aβ plaque analysis methods for mouse brain, but there is no such a study of analyzing the whole rat brain. Liebmann et al. (2016) proposed a pipeline for 3D study of AD pathologies in mouse brain hemispheres and human brain sections. They have demonstrated that tissue clearing and immunostaining of large samples enables high-throughput quantitation of complicated 3D-pathological features. They mainly focused on the individual plaque properties rather than the brain-wide plaque distribution. Whitesell et al. (2019) constructed a pipeline for mapping the spatial patterns of Aβ plaques in the whole mouse brain. Spatial patterns of Aβ deposits in the whole mouse brains were compared extensively among three transgenic animal lines at different ages. However, the dye methoxy-X04 could only label the dense-core plaques which might underestimate the plaque density. Nguyen et al. proposed a supervised learning method for quantifying Aβ plaques in the whole mouse brain with the random forest algorithm (Nguyen et al., 2019). This ilastik-based supervised learning method trained with few training data is not suitable for large-scale 3D whole-brain images with high dynamic range. Our deep learning-based method benefited from the rich semantic features extracted from the deep neural network is capable of analyzing the high-dynamic-range 3D whole brain images. Meanwhile, only a small size labeled training dataset is needed for network training makes deep learning-based analysis of the Aβ plaques in the whole brain more accessible.

Through this study, we have developed a systematic toolset for high-resolution imaging of the whole rat brain after labeling by pathological biomarkers. This toolset achieves high accuracy and requires lower consumption for the segmentation of immuno-labeled objects in 3D microscopic images and quantitative analysis of such objects by brain areas. We demonstrated the method with the first ever Aβ distribution atlas of the whole rat brain. Since this weakly supervised method greatly reduces the cost of manual labeling and the segmentation performance is almost state-of-the-art, this method could be commonly used in the segmentation and quantification of objects labeled with different biomarkers, such as cell bodies and protein aggregates.

## Data availability statement

The original contributions presented in this study are included in this article/Supplementary material, further inquiries can be directed to the corresponding authors.

## Ethics statement

The animal study was reviewed and approved by the Institutional Animal Care and Use Committee of Tsinghua University.

## Author contributions

ZC and HW designed the study, analysis method, and pipeline, and wrote the manuscript with inputs from WZ, KP, DX, LG, XC, and FW. ZC, WZ, XC, and FW architected the segmentation method. ZC performed the quantitative analysis of the data. KP proposed the study and provided the brain sample. ZC and DX performed the data labeling. ZC, HW, and LG prepared the sample and acquired the data. HW conceived and supervised the study. All authors contributed to the article and approved the submitted version.

## Footnotes

1
https://www.nitrc.org/projects/whs-sd-atlas
