# Supplementary material for: Weakly supervised learning analysis of Aβ plaque distribution in the whole rat brain
Source: Front Neurosci. 2023 Jan 19;16:1097019. doi: 10.3389/fnins.2022.1097019 (PMC9892753; doi:10.3389/fnins.2022.1097019)
Supplement: Supplementary file 1 [file Data_Sheet_1.PDF]

frontiers

# *Supplementary Material*

## 1 Supplementary Figures and Tables

### 1.1 Supplementary Figures

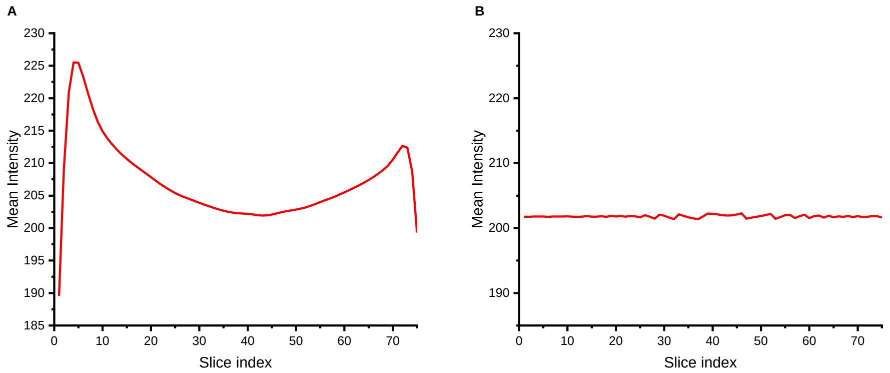


**Supplementary Figure 1.** Brightness calibration of the brain slices. (A) Example of a brain slice showing the mean intensity of the signal channel along z-axis of the section. (B) showing the mean intensity of the same slice after brightness calibration.

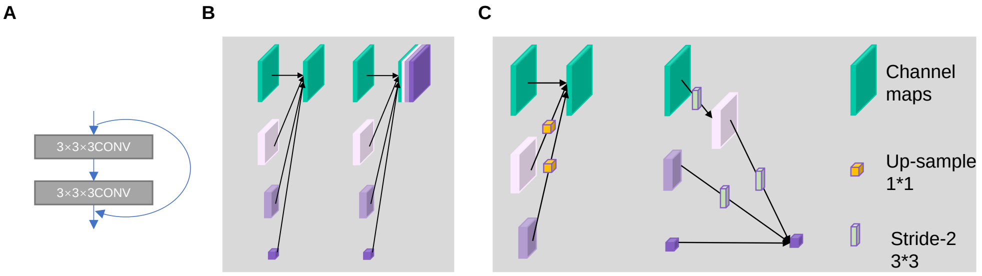


**Supplementary Figure 2.** Details of the HRNet. (A) the basic residual block. (B) left and right showing the HR-Keep mode and the HR-Fuse mode, respectively. (C) showing the architecture of the fuse module and the transition module from left to right.

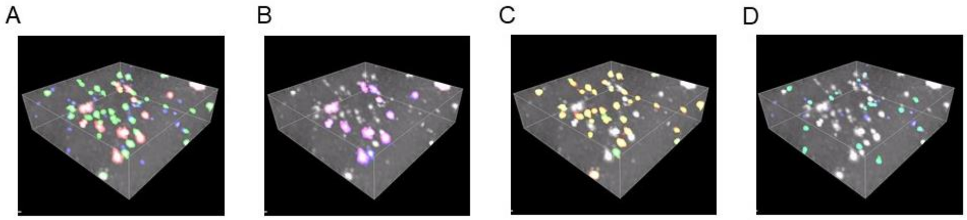


**Supplementary Figure 3.** Segmentation performance of plaques in different sizes. (A) The ground truth (GT). Red: big; Green: medium; Blue: small; (B) Big size. Red: GT; Blue: our prediction; (C) Medium size. Green: GT; Red: our prediction; (D) Small size. Blue: GT; Green: our prediction;

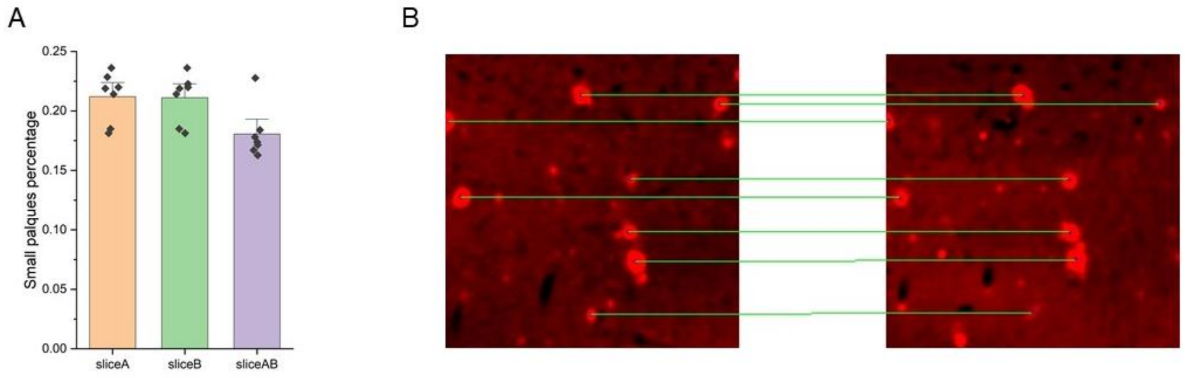


**Supplementary Figure 4.** The degree of signal matching. (A) Small plaques percentage of the total plaques in inner-sections and inter-sections. (B) The signal matching of spliced plaques in the adjacent sections.

## 1.2 Supplementary Tables

**Supplementary Table 1.** Performance comparison of different segmentation methods.

| Method | Label | DSC | | | SST | | | HD95($log_2$) | | |
|---|---|---|---|---|---|---|---|---|---|---|
| | | Cortex | Hippo | Other | Cortex | Hippo | Other | Cortex | Hippo | Other |
| Ilastik | Hand craft | 0.2535 | 0.3693 | 0.1522 | 0.1456 | 0.2285 | 0.0858 | 5.0811 | 5.4923 | 6.7476 |
| Segmenter-PMP34 | | 0.6404 | 0.445 | 0.1961 | 0.4784 | 0.2882 | 0.1129 | **2.2043** | 5.1346 | 6.8710 |
| U-Net | Fully | 0.6402 | 0.6102 | 0.3562 | 0.5365 | 0.5489 | 0.4996 | 4.5780 | 5.1645 | 5.6282 |
| HRNet | | 0.6087 | 0.5639 | 0.4859 | 0.4578 | 0.4161 | 0.4512 | 3.8624 | 4.8486 | 5.5778 |
| U-Net3D_rect | Weakly | 0.6948 | 0.4181 | 0.4206 | 0.8442 | 0.9149 | 0.6859 | 2.8464 | 4.5056 | 5.4748 |
| U-Net3DGrabcut | | 0.7434 | 0.4727 | 0.3781 | 0.6151 | **0.9257** | **0.7528** | 3.9749 | 4.5913 | 5.8066 |
| HRNet-fuse + OTSU-2D + PRM | | **0.7848** | **0.7428** | **0.5726** | **0.8386** | 0.8072 | 0.7379 | 3.1210 | **3.8112** | **5.3352** |

**Supplementary Table 2.** Ablation study of our method.

| Method | DSC | | | SST | | | HD95($log_2$) | | |
|---|---|---|---|---|---|---|---|---|---|
| | Cortex | Hippo | Other | Cortex | Hippo | Other | Cortex | Hippo | Other |
| CNN + OTSU | 0.5869 | 0.5326 | 0.4945 | 0.4284 | 0.3931 | 0.4287 | 5.0886 | 5.5597 | 6.0759 |
| CNN + OTSU-2D | 0.7169 | 0.6226 | 0.5177 | 0.6817 | 0.5862 | 0.5472 | 4.8409 | 5.3612 | 6.0266 |
| CNN + OTSU-2D + PRM | 0.7422 | 0.6415 | 0.5395 | 0.7278 | 0.6676 | 0.6139 | 4.2292 | 4.7393 | 5.7544 |
| HRNet-keep + OTSU | 0.5867 | 0.6188 | 0.4921 | 0.4309 | 0.4754 | 0.4513 | 4.5431 | 5.0567 | 6.0609 |
| HRNet-keep + OTSU-2D | 0.7473 | 0.7301 | 0.5444 | 0.6949 | 0.6971 | 0.5753 | 4.2838 | 4.8871 | 5.9910 |
| HRNet-keep + OTSU-2D + PRM | 0.7823 | 0.749 | 0.5703 | 0.7107 | 0.7094 | 0.6198 | 3.5918 | 4.2368 | 5.5382 |
| HRNet-fuse + OTSU | 0.6305 | 0.6184 | 0.5048 | 0.4912 | 0.5146 | 0.519 | 4.4898 | 4.6516 | 5.7131 |
| HRNet-fuse + OTSU-2D | 0.7597 | 0.7091 | 0.5472 | 0.801 | 0.7487 | 0.6612 | 3.9736 | 4.3158 | 5.6567 |
| HRNet-fuse + OTSU-2D + PRM | **0.7848** | **0.7428** | **0.5726** | **0.8386** | **0.8072** | **0.7379** | **3.1210** | **3.8112** | **5.3352** |

**Supplementary Table 3.** Performance comparison of different plaque sizes.

| Method | Label | DSC | | | SST | | | HD95($log_2$) | | |
|---|---|---|---|---|---|---|---|---|---|---|
| | | Small | Medium | Big | Small | Medium | Big | Small | Medium | Big |
| Ilastik | Hand craft | 0.0094 | 0.1658 | 0.4243 | 0.0048 | 0.0938 | 0.2754 | 7.3179 | 5.8349 | 3.7980 |
| Segmenter-PMP34 | | 0.2461 | 0.4359 | 0.5352 | 0.1715 | 0.3062 | 0.3763 | 6.8298 | 5.2265 | 4.1666 |
| U-Net | Fully | 0.0829 | 0.5039 | 0.7073 | 0.0956 | 0.4438 | 0.6940 | 6.7307 | 4.9761 | 3.8520 |
| HRNet | | 0.1581 | 0.5780 | 0.6296 | 0.1241 | 0.4897 | 0.4833 | 6.9968 | 4.3009 | **3.6113** |
| U-Net3D_rect | Weakly | 0.2104 | 0.5594 | 0.5379 | 0.2493 | 0.7772 | **0.9311** | 6.6327 | 4.5835 | 4.4225 |
| UNet3DGrabcut | | 0.1690 | 0.5552 | 0.6368 | 0.2812 | 0.6761 | 0.8584 | 6.5232 | 4.7163 | 4.0904 |
| HRNet-fuse + OTSU-2D + PRM | | **0.4281** | **0.7324** | **0.7779** | **0.4813** | **0.8208** | 0.8311 | **6.3301** | **3.8948** | 3.6364 |

**Supplementary Table 4.** Performance comparison of different plaque sizes in different brain regions.

| Method | DSC | | | SST | | | HD95($log_2$) | | |
|---|---|---|---|---|---|---|---|---|---|
| | Cortex | Hippo | Other | Cortex | Hippo | Other | Cortex | Hippo | Other |
| Small | 0.4735 | 0.4427 | 0.3711 | 0.4788 | 0.4354 | 0.5205 | 6.1753 | 6.3953 | 6.4207 |
| Medium | 0.7841 | 0.7642 | 0.6551 | 0.8280 | 0.8334 | 0.8035 | 3.2531 | 3.4651 | 4.5411 |
| Big | 0.8132 | 0.7711 | 0.7181 | 0.8796 | 0.8504 | 0.7031 | 2.0114 | 2.6077 | 5.2996 |
